# Effectiveness of evidence-based medicine training for undergraduate students at a Chinese Military Medical University: a self-controlled trial

**DOI:** 10.1186/1472-6920-14-133

**Published:** 2014-07-04

**Authors:** Xiangyu Ma, Bin Xu, Qingyun Liu, Yao Zhang, Hongyan Xiong, Yafei Li

**Affiliations:** 1 Department of Epidemiology, College of Preventive Medicine, Third Military Medical University, Chongqing 400038, People’s Republic of China; 2 Center for Clinical Epidemiology and Evidence-based Medicine, Third Military Medical University, Chongqing People’s Republic of China

**Keywords:** Evidence-based medicine, Education, Medical, Evaluation

## Abstract

**Background:**

To evaluate the effect of the integration of evidence-based medicine (EBM) into medical curriculum by measuring undergraduate medical students’ EBM knowledge, attitudes, personal application, and anticipated future use.

**Methods:**

A self-controlled trial was conducted with 251 undergraduate students at a Chinese Military Medical University, using a validated questionnaire regarding the students’ evidence-based practice (EBP) about knowledge (EBP-K), attitude (EBP-A), personal application (EBP-P), and future anticipated use (EBP-F). The educational intervention was a 20-hour EBM course formally included in the university’s medical curriculum, combining lectures with small group discussion and student-teacher exchange sessions. Data were analyzed using paired t-tests to test the significance of the difference between a before and after comparison.

**Results:**

The difference between the pre- and post-training scores were statistically significant for EBP-K, EBP-A, EBP-P, and EBP-F. The scores for EBP-P showed the most pronounced percentage change after EBM training (48.97 ± 8.6%), followed by EBP-A (20.83 ± 2.1%), EBP-K (19.21 ± 3.2%), and EBP-F (17.82 ± 5.7%). Stratified analyses by gender, and program subtypes did not result in any significant changes to the results.

**Conclusions:**

The integration of EBM into the medical curriculum improved undergraduate medical students’ EBM knowledge, attitudes, personal application, and anticipated future use. A well-designed EBM training course and objective outcome measurements are necessary to ensure the optimum learning opportunity for students.

## Background

Evidence-based medicine (EBM) encourages the assimilation and implementation of the conscientious, explicit, and judicious usage of currently best evidence in making decisions about the care of individual patients [1]. Since EBM was first defined by David Sackett, it is no longer considered acceptable for medical practice to be largely based on clinical anecdotes or expert opinions [2]. Rather, medical decisions should now be based on the best available evidence. EBM is recognized today as a practice that should be learned and applied by all physicians, epidemiologists, and medical administrators worldwide. Furthermore, evidence-based practice (EBP) is also used in low- and middle-income countries [3]. The acquisition of EBM knowledge and skills is also becoming recognized as a core competency that must be acquired by all doctors and medical students. Therefore, an increasing emphasis on training in EBM skills in undergraduate, postgraduate, and continuing medical education programs is becoming more widespread, and implemented, for example via traditional lectures, journal clubs, seminars, and e-learning classes [4-9].

EBM training should be evaluated and guided by evidence of its own effectiveness, including the awareness, attitudes, and competencies regarding EBM of medical students as well as in general practice. Despite the increasing number of medical schools and postgraduate programs that have introduced EBM in their curricula, no studies have evaluated the effectiveness of EBM training in China, especially for undergraduate students. Because educational research to support teaching and learning is important and the assessment and evaluation of new approaches is essential for medical education, in this study a self-controlled trial was performed using a validated questionnaire towards the knowledge (EBP-K), attitude (EBP-A), personal application (EBP-P), and future anticipated use (EBP-F) of EBM.

## Methods

### Study subjects and design

The Third Military Medical University, which affiliates to the People’s Liberation Army (PLA), is a Chinese military institution of higher learning and trains career physicians for the national military healthcare system. It offers 5- and 8-year programs, in which students receive bachelor and medical doctoral degrees on graduation, respectively. Included in this study were 251 students who had finished the premedical education and clinical courses (Additional file 1). A self-controlled study design was implemented to evaluate the teaching effects. The strategy was approved by the ethics committee of Third Military Medical University (reference number: IRB-TMMU-2012188), and the study was carried out in compliance with the Helsinki Declaration. A written informed consent (Additional file 2) was obtained from all participants which are all adults.

### Educational intervention and assessments

The educational intervention was a 20-hour EBM course formally included in the medical curriculum, including 5 lectures and 2 seminars. First, before the EBM lectures, students were asked to formulate answerable clinical questions based on common clinical scenarios. For example: (1) questions concerning the ability of a test to predict the likelihood of a disease (diagnosis); (2) questions concerning the effectiveness of a treatment or preventative measure (therapy); (3) questions concerning the likelihood of many factors coming to cause an illness (etiology); (4) questions concerning outcome of a patient with a particular condition (prognosis). Second, large group interactive lectures were conducted. The students were trained to develop search strategies according to the PICO format (Patient or Problem, Intervention, Comparison, and Outcome) and to comprehensively retrieve medical databases, including Pubmed, Medline, Embase, Cochrane library, and Web of Science. Teachers then demonstrated how to critically appraise, synthetize, and apply the evidence. Third, small group discussion sessions and student-teacher exchanges were conducted after each lecture. We evaluated the teaching effects of the interventions using validated instruments for the assessment of EBP in terms of knowledge (EBP-K), attitudes (EBP-A), personal application (EBP-P), and anticipated future use (EBP-F), which were employed before and after the EBM training [10]. The assessment questionnaires consisted of 26 questions modified from a reliable assessment tool using a 6-point Likert scale (Additional file 3) [11]. The EBP-K section included 5 items (scores: 5–30), the EBP-A and EBP-P sections 6 items each (scores: 6–36), and there were 9 items regarding EBP-F (scores: 9–54). This questionnaire was developed and validated for the assessment of EBM education in the undergraduate learning environment (with similar ages and backgrounds as our student participants) at the University of Hong Kong [11]. The scores in each category were summed for comparisons in the controlled trial.

### Statistical analyses

All statistical analyses were conducted using SAS version 9.2 (SAS Institute, Cary, NC). Mean scores, percentage change, and standard deviations (SD) were calculated to describe the differences for EBP-K, EBP-A, EBP-P, and EBP-F at the pre- and post-stages. In the before and after comparison, paired t-tests were used to determine the significance of the difference. All statistical tests were 2-tailed, and *P* < 0.05 was interpreted as statistically significant.

## Results

Totally there were 251 undergraduate students participated in this interventional study, completing both the pre- and post-training surveys (response rate = 100%). Of the 251 students, 21 are female (8.37%) and 230 were male (91.63%). Their ages ranged from 20 to 25, with a mean of 22 (SD = 1). Twenty-eight (11.16%) students were enrolled in the 8-year program and 223 (88.84%) were in the 5-year program. When investigated, they have the similar schedule of medical courses.

Table 1 presents the comparison of scores for the pre- and post-training surveys. Statistical significance for the difference between the pre- and post-training scores was detected for all four terms (***P*** **< 0.0001**). For EBP-K, EBP-P, and EBP-F, the summed post-training scores increased significantly, which means improvements in knowledge, personal application, and anticipated future use. The decreased score for EBP-A represents an improvement in the attitude towards EBM.

**Table 1:** Comparison of scores between survey ahead of the training and survey after the training

**Items**	Pre-scores*	Post-scores*	***P*** **Value**
EBP-K	21.57 ± 2.99	25.59 ± 4.80	**<0.0001**
EBP-A	12.91 ± 5.55	10.08 ± 6.28	**<0.0001**
EBP-P	14.23 ± 5.86	20.31 ± 5.66	**<0.0001**
EBP-F	39.56 ± 6.47	44.85 ± 7.97	**<0.0001**

*pre-scores refer to the scores ahead of the training; post-scores refer to the scores after the training.

**Figure 1:**
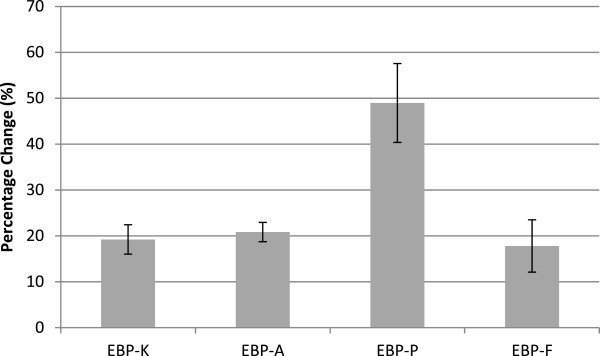
Percentage Changes of scores by EBM Training Course.

As shown in Figure 1, the most pronounced percentage change of scores after an EBM training course was EBP-P (48.97 ± 8.6%), followed by EBP-A (20.83 ± 2.1%), EBP-K (19.21 ± 3.2%), and EBP-F (17.82 ± 5.7%). Furthermore, stratified analyses were conducted. The percentage change of scores, were similar when stratified by gender, and program subtypes (Table 2).

**Table 2:** stratified comparisons of percentage change of scores before and after an EBM training course

	**Percentage**_**EBP-K**_	**Percentage**_**EBP-A**_	**Percentage**_**EBP-P**_	**Percentage**_**EBP-F**_
**Total**	19.21 ± 3.2%	20.83 ± 2.1%	48.97 ± 8.6%	17.82 ± 5.7%
**Gender**				
Male	19.23 ± 2.2%	20.84 ± 2.8%	48.96 ± 7.9%	17.83 ± 1.7%
Female	19.18 ± 1.8%	20.82 ± 2.2%	48.99 ± 8.4%	17.88 ± 6.1%
*P* value	0.920	0.200	0.414	0.925
**Program subtypes**				
Five-year	19.25 ± 2.8%	20.82 ± 1.7%	48.95 ± 7.6%	17.80 ± 5.4%
Eight-year	19.01 ± 3.4%	20.86 ± 1.1%	49.11 ± 9.5%	17.85 ± 4.8%
*P* value	0.508	0.094	0.528	0.283

## Discussion

To the best of our knowledge, this study represents the first large interventional trial regarding EBM training conducted in mainland China. The results indicate that significant improvements could be achieved in terms of knowledge, attitudes, personal application skills, and future use in clinical practice through EBM courses for upperclassmen who have completed their premedical education and clinical courses. These results indicate the importance of EBM training for undergraduates, and demonstrate that undergraduate training may serve as an active and useful measure to bridge the gap between medical students and doctors and foster the implementation of EBM.

Considering the high impact of EBP, physicians are increasingly being encouraged to apply EBM. Worldwide, numerous initiatives have emerged to promote EBM including traditional lectures, journal clubs, seminars, workshop, e-learning classes, and competitions [12-18]. However, most of the previous interventions were performed among physicians, nurses, or other clinical staff, and very few were conducted with undergraduates [19]. Although some medical schools have included the principles of EBM as a subject within their medical curriculum, few studies have explored the barriers and enablers that students experience when studying medicine and attempting to integrate EBM into their clinical experience. The principles and application of EBM are perceived to be important for medical students, in both their current clinical training and future work as clinicians. Thus, the university restructured its curriculum and included the EBM course, which emphasized EBM practices and aimed to improve students’ practical application of the tool.

EBP is the integration of the best research evidence with patients’ values and clinical circumstances in clinical decision making, thus teaching EBM should be evaluated and guided by evidence of its own effectiveness [20]. More than 100 unique assessment strategies that target different objectives have been developed [20]. In this study we considered a number of instruments with reasonable validity that could evaluate some domains of EBP and target different evaluation needs. We finally chose the questionnaire developed by Johnston et al. [10]. The survey focused on the evaluation of EBP knowledge, attitude, personal application, and anticipated future use, and the questionnaire was developed and validated for the assessment of EBM education in students with similar backgrounds.

In the current study, EBM knowledge, attitudes, personal application, and anticipated future use increased in different degrees. In contrast to a previous study in which EBP-K showed the greatest change across the training [11], our study found that EBP-P had the most pronounced percentage change of scores after EBM training (48.97 ± 8.6%). This is because of the training’s emphasis on application skills and EBP methods, and as such it is now viewed as an achievement of our EBM teaching reform. The practice of EBM depends more on application skills and methodology (in clinical studies) than on detailed knowledge. The EBP-P scores in our study are also higher than those in Cheng’s study [11]. This reveals the effectiveness of practical training in EBM skills.

## Conclusion

In summary, the integration of EBM in a medical curriculum improved the EBM knowledge, attitudes, personal application, and anticipated future use of such methods in a sample of 251 undergraduate medical students. A well-designed EBM training program and objective outcome instruments are necessary to ensure the effective implementation and measurement of such methods. A long-term follow-up on the effects of EBM skills on patient outcomes in future studies will further strength and validate the conclusion of our research.

## Competing interests

The authors declare that they have no conflicts of interest. The authors alone are responsible for the content and writing of the paper.

## Authors’ contributions

MX, XB, LQ, ZY collected the data, undertook statistical analysis and drafted the manuscript. LY, XH, MX developed the study protocol. All authors read and approved the final manuscript.

## Authors’ information

Xiangyu Ma, MD, PhD, is an instructor of evidence-based medicine education at the third military medical university.

Bin Xu, MD, MPH, is an instructor of evidence-based medicine education at the third military medical university.

Qingyun Liu, MD, is a master student at the third military medical university.

Yao Zhang, MD, PhD, is an associate professor of evidence-based medicine education.

Hongyan Xiong, MD, PhD, is a professor of evidence-based medicine education.

Yafei Li, MD, PhD, is a professor and the director of evidence-based medicine education.

## Pre-publication history

The pre-publication history for this paper can be accessed here:

http://www.biomedcentral.com/1472-6920/14/133/prepub

## Supplementary Material

Additional file 1 The finished the premedical education and clinical courses. Click here for file

Additional file 2 Consent to participate “Evidence-Based Medicine Training” Project. Click here for file

Additional file 3 Questionnaires of EBM skills. Click here for file
