# Supplementary material for: Effectiveness of evidence-based medicine training for undergraduate students at a Chinese Military Medical University: a self-controlled trial
Source: BMC Med Educ. 2014 Jul 4;14:133. doi: 10.1186/1472-6920-14-133 (PMC4091652; doi:10.1186/1472-6920-14-133)
Supplement: Additional file 1 — The finished the premedical education and clinical courses. [file 1472-6920-14-133-S1.docx]

**Additional file 1: The finished the premedical education and clinical courses**

**Premedical courses**

Psychology human anatomy, histology and embryology, physiology, biochemistry, cell biology, microbiology, parasitology, immunology, statistics, medical genetics, pathology, physiology pharmacology, pathology

**Clinical courses**

Surgery Pandect and operation, diagnosis, internal medicine, surgery, gynecology and obstetrics, pediatrics, Oral medicine, otolaryngology, neurology, reproductive medicine, rehabilitation medicine, dermatology and Venereology, infectious diseases, critical care, emergency medicine, Ophthalmology
