# Supplementary material for: Effectiveness of evidence-based medicine training for undergraduate students at a Chinese Military Medical University: a self-controlled trial
Source: BMC Med Educ. 2014 Jul 4;14:133. doi: 10.1186/1472-6920-14-133 (PMC4091652; doi:10.1186/1472-6920-14-133)
Supplement: Additional file 2 — Consent to participate “Evidence-Based Medicine Training” Project. [file 1472-6920-14-133-S2.docx]

**Additional file 2: Consent to participate “Evidence-Based Medicine Training” Project**

Introduction:

The acquisition of EBM knowledge and skills is also becoming recognized as a core competency that must be acquired by all doctors and medical students. Also, EBM training should be evaluated and guided by evidence of its own effectiveness, including the awareness, attitudes, and competencies regarding EBM of medical students as well as in general practice. Despite the increasing number of medical schools and postgraduate programs that have introduced EBM in their curricula, no studies have evaluated the effectiveness of EBM training in China, especially for undergraduate students.

Thus, we need your participation. As a participant in my project, you will:

1) receive an educational intervention of 20-hour EBM course formally included in the medical curriculum, including 5 lectures and 2 seminars;

2) complete questionnaires consisted of 26 questions modified from a reliable assessment tool using a 6-point Likert scale.

Questionnaire results will be reported a group summary and not individually tracked. Participant responses will be anonymous. There are no physical or mental risks to your participation in the project.

Thank you for helping us in this special way,

Yafei Li, Xiangyu Ma

**Individual Consent Statement**

I agree to participate in the " Evidence-Based Medicine Training " project. I understand that any information I provide will be anonymous and not individually tracked. A summary of the project is available to me upon request.

Signature:

Date:
